# A citizen science-based survey of avian mortality focusing on haemosporidian infections in wild passerine birds

**DOI:** 10.1186/s12936-021-03949-y

**Published:** 2021-10-23

**Authors:** Tanja Himmel, Josef Harl, Julia Matt, Herbert Weissenböck

**Affiliations:** Institute of Pathology, Department for Pathobiology, University of Veterinary Medicine, Veterinärplatz 1, 1210 Vienna, Austria

**Keywords:** Citizen science, Dead bird, *Plasmodium*, *Haemoproteus*, *Leuocytozoon*, Exo-erythrocytic merogony, In situ hybridization, Avian malaria, Haemosporidioses

## Abstract

**Background:**

Haemosporidioses are common in birds and their manifestations range from subclinical infections to severe disease, depending on the involved parasite and bird species. Clinical haemosporidioses are often observed in non-adapted zoo or aviary birds, whereas in wild birds, particularly passerines, haemosporidian infections frequently seem to be asymptomatic. However, a recent study from Austria showed pathogenic haemosporidian infections in common blackbirds due to high parasite burdens of *Plasmodium matutinum* LINN1, a common parasite in this bird species, suggesting that virulent infections also occur in natural hosts. Based on these findings, the present study aimed to explore whether and to what extent other native bird species are possibly affected by pathogenic haemosporidian lineages, contributing to avian morbidity.

**Methods:**

Carcasses of passerine birds and woodpeckers were collected during a citizen science-based survey for avian mortality in Austria, from June to October 2020. Tissue samples were taken and examined for haemosporidian parasites of the genera *Plasmodium*, *Haemoproteus* and *Leucocytozoon* by nested PCR and sequencing the mitochondrial *cytb* barcode region, histology, and chromogenic in situ hybridization applying genus-specific probes.

**Results:**

From over 160 dead bird reportings, 83 carcasses of 25 avian species were submitted for investigation. Overall haemosporidian infection rate was 31%, with finches and tits prevailing species counts and infections. Sequence analyses revealed 17 different haplotypes (4 *Plasmodium*, 4 *Haemoproteus*, 9 *Leucocytozoon*), including 4 novel *Leucocytozoon* lineages. Most infected birds presented low parasite burdens in the peripheral blood and tissues, ruling out a significant contribution of haemosporidian infections to morbidity or death of the examined birds. However, two great tits showed signs of avian malaria, suggesting pathogenic effects of the detected species *Plasmodium relictum* SGS1 and *Plasmodium elongatum* GRW06. Further, exo-erythrocytic tissue stages of several haemosporidian lineages are reported.

**Conclusions:**

While suggesting generally little contribution of haemosporidian infections to mortality of the investigated bird species, the findings indicate a possible role of certain haemosporidian lineages in overall clinical manifestation, either as main causes or as concurrent disease agents. Further, the study presents new data on exo-erythrocytic stages of previously reported lineages and shows how citizen science can be used in the field of haemosporidian research.

**Graphic abstract:**

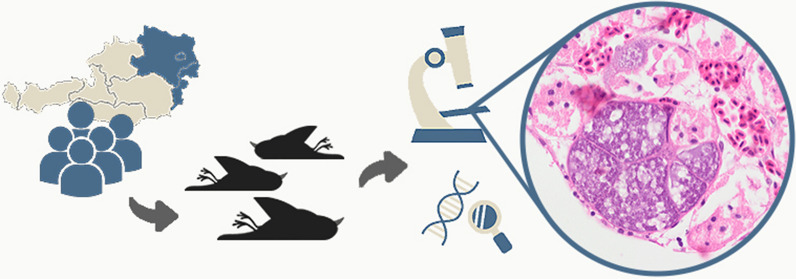

**Supplementary Information:**

The online version contains supplementary material available at 10.1186/s12936-021-03949-y.

## Background

Avian haemosporidians of the genera *Plasmodium*, *Haemoproteus*, and *Leucocytozoon* are arthropod-transmitted blood parasites with almost global distribution, infecting birds of diverse phylogenetic orders [1]. Counting more than 250 morphospecies [1, 2] and over 4500 genetic lineages in the MalAvi database (http://130.235.244.92/Malavi/ [3]), these protozoans vary considerably in their host specificity and pathogenicity [4, 5]. While generally considered benign in most birds, avian haemosporidia can cause severe clinical diseases and mortality in susceptible hosts [1, 6]. Birds without previous parasite exposure are particularly vulnerable and may experience severe parasitaemia during acute infections, resulting in haemolysis, anaemia, and death [7–9]. Birds may also die without premonitory signs, and fatalities are often linked to deleterious effects of exo-erythrocytic parasite stages in internal organs [10, 11].

Reports of clinical haemosporidioses frequently refer to domesticated or exotic bird species kept outside of their geographic origin such as in zoos or aviaries. In zoological settings, captive birds often live in close contact to the indigenous avifauna, which serves as a reservoir for haemosporidian parasites [9]. Transmission of the parasites to immunologically naïve bird hosts can result in increased virulence of otherwise non-pathogenic lineages, sometimes leading to disease outbreaks with high mortality rates, as, for example, regularly observed in *Plasmodium*-infected penguins [12] or *Haemoproteus*-infected parakeets [13]. In natural bird populations, haemosporidian disease outbreaks have been associated with the introduction of new lineages to naive host communities [14]. The probably best known example for the negative impact of haemosporidian infection on hosts without co-evolutionary adaption are Hawaiian honeycreepers, which have suffered severe population declines due to avian malaria after the introduction of both *Plasmodium relictum* GRW4 and its mosquito vector *Culex quinquefasciatus* to the islands around 1900 [14–16]. Similarly, in New Zealand, the introduction or translocation of avian hosts, together with the spread of invasive mosquito species, has led to multiple mortality events caused by avian malaria in several endemic bird species [17].

Apart from these well-documented cases of fatal haemosporidioses in naïve bird populations, little is known about Haemosporida-associated diseases and pathogenicity in wild birds inhabiting regions with local haemosporidian transmission. Field studies show that free-ranging birds often appear as asymptomatic carriers with low parasitaemia, suggesting subtle health effects of chronic infections in adapted hosts. Some studies revealed correlations of infections with increased physiological stress [18], reduced survival [19] and shorter lifespans [20], however, other studies yielded more ambiguous results [21, 22]. Besides consequences for host fitness, fatal haemosporidian infections have been described in a range of wild bird species, including gallinaceous birds [23], buzzards [24], falcons [25], different passerines [26–31], frogmouths [28], owls [32], woodpeckers [33], pigeons [34], and penguins [35, 36]. In addition to these mostly incidental reports in naturally infected birds, lethal haemosporidian infections were demonstrated in several experimentally infected passerines, providing indisputable evidence for the pathogenicity of certain lineages in wild birds [37].

It is important to note that in haemosporidian field studies, techniques for collecting free-ranging birds, such as the use of mist nets, may favour the capture of subclinically infected individuals, as sick birds might exhibit reduced mobility [38, 39]. In contrast, the detection of diseased or dead birds in the natural environment is problematic due to their rapid decomposition or removal by predators [40, 41], potentially resulting in biased sampling towards healthy birds. Considering these detection difficulties, it is conceivable that severe haemosporidioses, particularly acute infections, are underreported in wild birds.

Wildlife disease monitoring strategies such as routine pathological investigations of *ad hoc* submitted carcasses are fundamental to detecting wildlife morbidity and mortality, particularly when clinical manifestations are less obvious. While these investigations provide important epidemiological data by determining the presence or absence of particular pathogens, and identifying causes of death, they are constrained by the submission of carcasses. Citizen science, the participation of the public community in scientific research, offers a suitable approach to wildlife disease monitoring and has been used for the surveillance of different avian pathogens, including highly pathogenic avian influenza virus [42], West Nile virus [41, 43], Usutu virus [44], *Salmonella typhimurium* [45, 46], *Mycoplasma gallisepticum* [47], or *Trichomonas gallinae* [48]. By involving volunteer participants (“citizen scientists”) in wildlife morbidity and mortality reporting, citizen science can facilitate the detection and recovery of carcasses of target species in both space and time, enabling large-scale epidemiological surveys and the collection of tissue samples [49].

Exploiting such archives of tissue samples, previous retrospective studies in Austria revealed an association of blackbird mortalities with the presence of widespread haemosporidian lineages [30, 50]. Notably, severe parasite loads were linked to infections with *Plasmodium matutinum* LINN1, pointing towards its pathogenic role in these birds’ mortalities. Single cases of virulent *Plasmodium* infections were also recorded in sparrows, tits, chaffinches, and woodpeckers [30], however, due to low sample sizes, it remains unclear to what extent these and other passerine species are affected by pathogenic lineages present in Austria. To address this question, the present study aimed to collect carcasses of passeriform and piciform birds by means of citizen science, and examine them for haemosporidian parasites.

## Methods

### Study area and collection of bird carcasses

For the collection of bird carcasses, a citizen science project was initiated in spring 2020. From June to October 2020, Austrian citizens were asked to report findings of dead birds and submit collected carcasses to the Institute of Pathology, University of Veterinary Medicine Vienna, for pathological examination. In order to reach a broad community of citizens, the survey was announced via different media prior to the start of the project, including a press release, local newspapers, radio interviews, and social media (Facebook). The survey was conducted in collaboration with the non-governmental organisation BirdLife Austria, and the urban wildlife project “StadtWildTiere”. Reportings of dead birds were received via emails, telephone, and a reporting form made available on the website of “StadtWildtiere”. In addition to the reports, citizen scientists sent photographs of the bird carcasses for species determination. *A priori* restrictions to the collection of dead birds included decomposed condition of the carcasses, and mortalities suspicious for finch trichomonosis. The dead birds were collected from all over Austria, however, for logistic reasons the geographic focus was on the Eastern federal states Vienna, Lower Austria and Burgenland. Citizen scientists were advised to store recovered bird carcasses in closed plastic bags at a cool place before their pick-up and transportation to the University of Veterinary Medicine Vienna by a medical courier service.

### Tissue sampling

Necropsy was performed on submitted carcasses and tissue samples were collected from heart, lung, liver, spleen, kidney, brain, skeletal muscle, proventriculus, gizzard, intestine, bursa of Fabricius, and tibiotarsus for examination of the bone marrow. For molecular analyses, fresh samples of liver, lung, and brain were frozen and stored at − 20 °C until further processing. For histopathology and chromogenic in situ hybridization (CISH), tissue samples were fixed in 10% neutral buffered formalin for 24 h before routine embedding in paraffin wax. Formalin-fixed paraffin-embedded tissues (FFPE) were cut at 1–2 μm and stained with haematoxylin and eosin (HE). Morphological identification of haemosporidian tissue stages in HE-stained sections was based on parasite identification keys presented in the review by Valkiūnas and Iezhova [51].

### Molecular analyses

Total DNA was extracted from frozen tissue samples using the DNeasy Blood & Tissue Kit (Qiagen, Venlo, Netherlands) according to the manufacturer’s instructions with one modification: the DNA was eluted twice with each 100 µl AE buffer, and the second eluate was used as template for the PCR. Molecular screening was done using a well-established nested PCR protocol targeting a partial sequence of the mitochondrial cytochrome b gene (*cytb*) of avian haemosporidians [52]. First, the outer primers HaemNFI and HaemNR3 were used to amplify DNA of *Plasmodium*, *Haemoproteus*, and *Leucocytozoon*. Second, the inner primer pairs HaemF/HaemR2 and HaemFL/HaemR2L were used to amplify DNA of *Plasmodium*/*Haemoproteus*, and *Leucocytozoon*, respectively. PCRs were performed in 25 µl reaction volumes containing 14.375 µl nuclease-free water, 5 µl 5X Green GoTaq Flexi Buffer (Promega, Madison, Wisconsin, USA), 2 µl MgCl_2_ solution (25 mM), 0.5 µl PCR nucleotide mix (10 mM, Promega), 0.125 µl GoTaq G2 Flexi DNA Polymerase (5 u/µl, Promega), each 1 µl forward and reverse primers (10 pmol/µl), and 1 µl DNA template or, in case of the second PCRs, 1 µl amplicon from the first PCR. All reactions were run initial 2 min at 94 °C, followed by 35 cycles of 30 s denaturation at 94 °C, 30 s primer annealing at 50 °C, and 1 min extension at 72 °C, followed by 10 min final extension at 72 °C. PCR success was checked by gel electrophoresis of the PCR products on 1% agarose gels with Midori Green Advance (Nippon Genetics Europe, Dueren, Germany) and visualization of amplicons using a BioSens SC-Series 710 gel documentation system (GenXpress, Wiener Neudorf, Austria). Tissue samples were processed along with positive controls (samples confirmed positive in previous screenings) and negative controls (nuclease-free water) in every PCR run. PCR amplicons from the second PCRs were sent to Microsynth Austria for bi-directional sequencing. Obtained sequences and electropherograms were analyzed with Bioedit [53]. Sequences with ambiguous characters, indicating multiple infections, were double checked and un-phased using DnaSP v.6.12.3 [54]. All sequences were subjected to BLAST search in the avian malaria database MalAvi (http://130.235.244.92/Malavi/ [3]) and NCBI GenBank. Haplotypes not matching 100% with previously published *cytb* lineages were assigned new lineage names according to MalAvi rules [3]. Nucleotide sequences of the detected haemosporidians were uploaded to GenBank under the accession numbers MZ465327– MZ465365.

In addition to Haemosporida-PCR screening, tissue samples of birds that showed macroscopic lesions suggestive for *Trichomonas* infection during necropsy were screened for the presence of *Trichomonas* parasites. For this purpose, esophagus and crop samples were subjected to DNA-extraction and PCR using previously established primers (forward primer 5′-GGT AGG CTA TCA CGG GTA AC-3′, reverse primer 5′-ACT YGC AGA GCT GGA ATT AC-3′), which target a 247-249 bp section of the 18 S ribosomal RNA gene of parasites belonging to the order Trichomonadida [55].

### Chromogenic in situ hybridization

To detect haemosporidian parasites in tissue sections, FFPE tissue blocks of PCR-positive birds were cut into 1–2 μm thick sections, one of which was stained with HE and the remaining were subjected to CISH. Chromogenic in situ hybridization was performed following previously established protocols using genus-specific oligonucleotide probes for the detection of *Haemoproteus* and *Leucocytozoon* [56]. For the detection of *Plasmodium*, an oligonucleotide probe was designed based on *18S* ribosomal RNA gene sequences of *Plasmodium* spp. [57]. In addition, species-specific probes were designed for the detection of *Plasmodium relictum* and *Plasmodium elongatum*. These probes were previously tested on tissues featuring single infections with the respective parasites, ruling out cross-reactions. For all cases with mixed infections, as determined by PCR and sequencing, several sections were separately incubated with the relevant probes. Sequences of all probes used in this study are provided in Table 1.

**Table 1:** Oligonucleotide probes used for the detection of avian haemosporidian parasites by chromogenic in situ hybridization

Probe	Sequence	Work concentration	Target	References
Plasmo18S_1	5′-CTTAAACTTCCTTGTGTTAGACACACAAT-3′	1 ng/100 µl	*Plasmodium* spp.	Present study
Haemo18S_1	5′-GCTAACCGTAGTTATAGTCGCCATCTC-3′	1 ng/100 µl	*Haemoproteus* (*Parahaemoproteus*) spp.	Himmel et al. [56]
Leuco18S_1	5′-TAGGACTCCCCACTTGTCTTTTTCTTGA-3′	1 ng/100 µl	*Leucocytozoon* spp.	Himmel et al. [56]
Prel18S	5′-ACCATTTAACACGTATCCGATAAAGCATTACC-3′	10 ng/100 µl	*Plasmodium relictum* (SGS1)	Present study
Pelo18S	5′-CAACTGTTACATTGGGACGCCTTT-3′	10 ng/100 µl	*Plasmodium elongatum* (GRW06)	Present study

CISH procedure was done as previously described in [30, 56]. In brief, tissue sections were deparaffinized, rehydrated in a series of graded ethanol (100%, 96%, 70%) and subjected to proteolytic treatment with proteinase K (Roche, Basel, Switzerland) for 40 min at 37 °C. Thereafter, sections were rinsed in distilled water, dehydrated in 96% and 100% ethanol and air-dried before incubation with hybridization solution containing 1 or 10 ng digoxigenin-labelled probe per 100 µl. Hybridization was done overnight in a humid chamber at 40 °C. On the following day, after stringency washes in 2X, 1X, 0.1X SSC buffer 10 min each, tissue sections were covered with blocking solution containing normal goat serum and 10% Triton X-100 for 30 min before application of anti-digoxigenin-AP Fab-fragments (Roche) at a concentration of 1:200 for 1 h at room temperature. After two washes in distilled water for 15 min, tissue sections were incubated with NBT/BCIP (nitro-blue tetrazolium chloride/5-bromo-4-chloro-3’-indolyphosphate p-toluidine salt, Roche) mixed with levamisole in 0.1 M Tris-buffered saline (pH 9.5) for minimum 40 min in a dark, humid chamber. After stopping the chromogenic reaction with Tris-EDTA buffer (pH 8.0) for 10 min, sections were counterstained with haematoxylin and mounted using Aquatex (Merck Millipore) and coverslips. All in situ hybridized sections were evaluated using brightfield microscopy and 100–400× magnifications. Based on the location, size, and shape of the chromogenic signal, a distinction was made between signals representing blood stages of the parasite and signals representing exoerythrocytic tissue stages. For example, signals found in the lumen of blood vessels measuring up to the size of erythrocytes were regarded as blood stages, whereas signals located in other host cells and exceeding the size of erythrocytes were regarded as exoerythrocytic meronts. Detailed descriptions and examples on how CISH signals were distinguished can be found in [50]. In addition, corresponding HE-stained sections were checked to confirm presence or absence of exoerythrocytic stages. To be able to compare infection intensities, the abundance of blood stage signals was scored for each case as low (0–3 signals in a blood vessel of approximately 100 μm in diameter), moderate (4–20 signals) or high (more than 20 signals).

## Results

### Dead bird reports

From June to October 2020 at least 166 reports counting more than 326 dead birds of 31 species and 17 families were received via website or email. As intended, the majority of reported dead birds were members of the order Passeriformes (296 individuals) and Piciformes (23). Seven dead birds belonged to the orders Columbiformes (3), Coraciiformes (1), Falconiformes (1), Pelecaniformes (1) and Galliformes (1). Among passeriform birds, finches (Fringillidae) were most frequently reported, particularly European greenfinches *Chloris chloris* with over 85 individuals, followed by common chaffinches *Fringilla coelebs* (29) and European goldfinches *Carduelis carduelis* (21). Other reported passeriform birds included species of Turdidae (30), Paridae (20), Passeridae (18), Hirundinidae (14), Muscicapidae (10), Sylviidae (3), Sturnidae (2), Corvidae (2), Regulidae (1) and Emberizidae (1). Among Piciformes, the great spotted woodpecker *Dendrocopos major* was most frequently reported with ten individuals, followed by the European green woodpecker *Picus viridis* with six individuals.

The majority of dead birds (over 75%) were registered between June and August 2020. As the geographic focus of the survey was on eastern Austria, about half of the reported birds were found in the federal state Lower Austria (154), followed by the capital Vienna (41) and Burgenland (30), however, reports were also received from more western federal states. Most birds were found on private property, either in the garden or around the house, for example on the balcony or terrace. A high percentage of the citizen scientists stated to feed birds regularly.

### Pathological examinations

In total, 83 individuals were collected by the citizen scientists and submitted for pathological examination. Due to high numbers of birds not suitable for investigation, the number of submitted carcasses was lower compared to reported dead bird counts. Two of the 83 submitted birds were not examined due to their advanced decomposition at the time of delivery. Except for these two individuals, which could only be identified to genus level, all birds were identified to species level. Among the submitted birds, great tits *Parus major* and common chaffinches *Fringilla coelebs* were the most common species with 10 and 9 individuals, respectively (Table 2). In 24 of 81 birds, gross examination showed signs of trauma from bird strikes (i.e. collision with structures such as windows), for example subdermal intracranial haemorrhages, crushes, or blood in the thoraco-abdominal cavity. In finches, gross lesions suggestive of avian trichomonosis such as yellowish and necrotic masses on the oral mucosa, oesophagus, or crop were frequently observed (see Additional file 1). Other abnormalities observed during necropsy included spleno- and hepatomegaly, and dark or bloody contents in the intestines.

**Table 2:** Haemosporidian parasites detected in dead birds submitted between June and October 2020 and screened by PCR

Bird family	Host species	*n*	Sample origin (Federal state)	PCR positive				Parasite species & *cytb* lineages^a^
				Plas	Haem	Leuc	Total (%)	
Passeridae	*Passer domesticus*	2	V (1), LA (1)	–	–	–	0 (0%)	
	*Passer montanus*	1	LA	–	–	–	0 (0%)	
	*Passer* sp.	2	LA	–	–	–	0 (0%)	
Fringillidae	*Carduelis carduelis*	6	LA (2), S (1), T (3)	1	–	–	1 (17%)	*P. vaughani* SYAT05
	*Chloris chloris*	7	V (2), LA (1), S (1), B (3)	–	1	–	1 (14%)	*Haemoproteus* sp. CARCHL01
	*Fringilla coelebs*	9	V (1), LA (6), S (1), B (1)	1	7	2	9 (100%)	*P. relictum* GRW11 (1), *Haemoproteus* sp. CCF23 (7), *Leucocytozoon* sp. BRAM3 (2)
	*Coccothraustes coccothraustes*	3	LA (3)	–	1	3	3 (100%)	*Haemoproteus* sp. HAWF6 (1), *Leucocytozoon* sp. HAWF7 (3), *Leucocytozoon* sp. **COCCOC01** (2), *Leucocytozoon* sp. **COCCOC02** (1)
	*Pyrrhula pyrrhula*	1	T	–	1	1	1 (100%)	*Haemoproteus* cf. *majoris* EMSPO03, *Leucocytozoon* sp. PARUS25, *Leucocytozoon* sp. BT2
	*Serinus serinus*	1	T	–	–	–	0 (0%)	
	*Linaria cannabina*	1	LA	–	–	–	0 (0%)	
Paridae	*Parus major*	10	V (4), LA (5), B (1)	4	–	1	4 (40%)	*P. elongatum* GRW06 (4), *P. relictum* SGS1 (2), *Leucocytozoon* sp. PARUS4 (1)
	*Cyanistes caeruleus*	3	V (2), UA (1)	1	–	2	3 (100%)	*P. elongatum* GRW06 (1), *Leucocytozoon* sp. PARUS4 (2), *Leucocytozoon* sp. **CYACAE07** (1)
Hirundinidae	*Hirundo rustica*	5	LA (5)	–	–	–	0 (0%)	
Sylviidae	*Sylvia atricapilla*	2	LA (1), B (1)	–	–	–	0 (0%)	
Regulidae	*Regulus regulus*	1	V	–	–	–	0 (0%)	
Muscicapidae	*Phoenicurus ochruros*	3	V (3), LA (1)	–	–	–	0 (0%)	
	*Erithacus rubecula*	3	LA (3)	–	–	–	0 (0%)	
Turdidae	*Turdus philomelos*	1	LA	–	–	–	0 (0%)	
	*Turdus merula*	2	LA (1), UA (1)	2	–	–	2 (100%)	*P. vaughani* SYAT05 (2)
Sturnidae	*Sturnus vulgaris*	3	V (3)	-	–	–	0 (0%)	
Picidae	*Picus viridis*	6	LA (5), B (1)	–	–	–	0 (0%)	
	*Dendrocopos major*	5	LA (3), B (2)	–	–	–	0 (0%)	
	*Leiopicus medius*	2	V (1), LA (1)	–	–	–	0 (0%)	
Alcedinidae	*Alcedo atthis*	1	B	–	–	–	0 (0%)	
Ardeidae	*Botaurus stellaris*	1	V	–	–	1	1 (100%)	*Leucocytozoon* sp. **BOTSTEL01**
Total		81		9	10	10	25	17 lineages (4 *Plas*, 4 *Haem*, 9 *Leuc)*

V: Vorarlberg; LA: Lower Austria; S: Salzburg; T: Tyrol; B: Burgenland; V = Vienna

Plas: *Plasmodium*; *Haem*: *Haemoproteus*; *Leuc*: *Leucocytozoon*

^a^Numbers in parentheses indicate total infected birds. Lineages in bold represent novel *cytb* haplotypes

### Haemosporidian prevalence and diversity

**Fig. 1:**
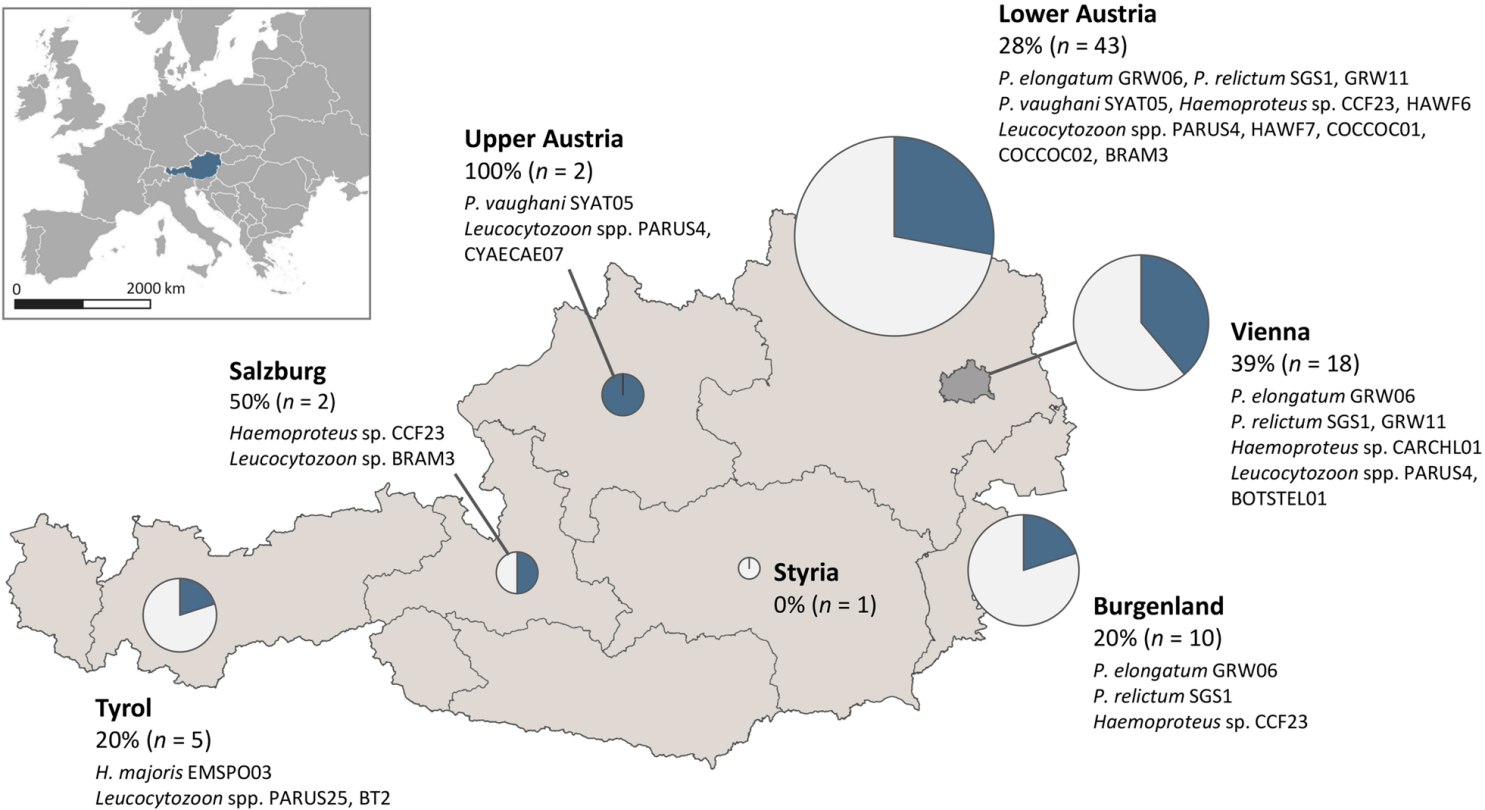
Geographic distribution of bird carcasses sampled in Austria (June-October 2020), proportion of infected birds, and detected haemosporidian ***cytb*** lineages. Pie charts represent sampling effort (circle size) in the different federal states and the percentage of haemosporidian-positive birds (blue slices)

By PCR, 25 (31%) of 81 submitted birds tested positive for haemosporidian parasites of the genera *Plasmodium*, *Haemoproteus* or *Leucocytozoon*, however the proportion of infected birds varied strongly between host species (Table 2). A 100% infection rate was found in common chaffinches *Fringilla coelebs* (n = 9), hawfinches *Coccothraustes coccothraustes* (n = 3), blue tits *Cyanistes caeruleus* (n = 3), blackbirds *Turdus merula* (n = 2), the bullfinch *Pyrrhula pyrrhula* (n = 1) and Eurasian bittern *Botaurus stellaris* (n = 1). Among great tits *Parus major *(n = 10) 40% of the birds were haemosporidian-positive, whereas European goldfinches *Carduelis carduelis* (n = 6) and European green finches *Chloris chloris* (n = 7) were less often infected with 17% and 14%, respectively. Among all examined birds, the three genera *Plasmodium*, *Haemoproteus* and *Leucocytozoon* were similarly prevalent with 9, 10, and 10 infected birds, respectively. Eight (32%) of the 25 positive birds showed mixed infection with parasites of the same genus (2) or different genera (5). By sequencing, 17 unique *cytb* lineages were identified, most of which were identical to haplotypes previously deposited in the MalAvi database or Genbank (Table 2). The majority of lineages were recorded in finches and tits, which accounted for half of all examined birds. For two known *cytb* lineages, new host-parasite associations were identified, including SYAT05, a lineage of *Plasmodium vaughani*, detected in a goldfinch *Carduelis carduelis*, and BT2 (*Leucocytozoon* sp.), detected in a bullfinch *Pyrrhula pyrrhula*. Four *Leucocytozoon* lineages (COCCO01, COCCOC02, CYACAE07, BOTSTEL01) were recorded for the first time in this study. Figure 1 shows the geographic distribution of all investigated birds and detected haemosporidian lineages. Detailed information for individual positive cases are provided in Additional file 1.

### Chromogenic in situ hybridization

**Fig. 2:**
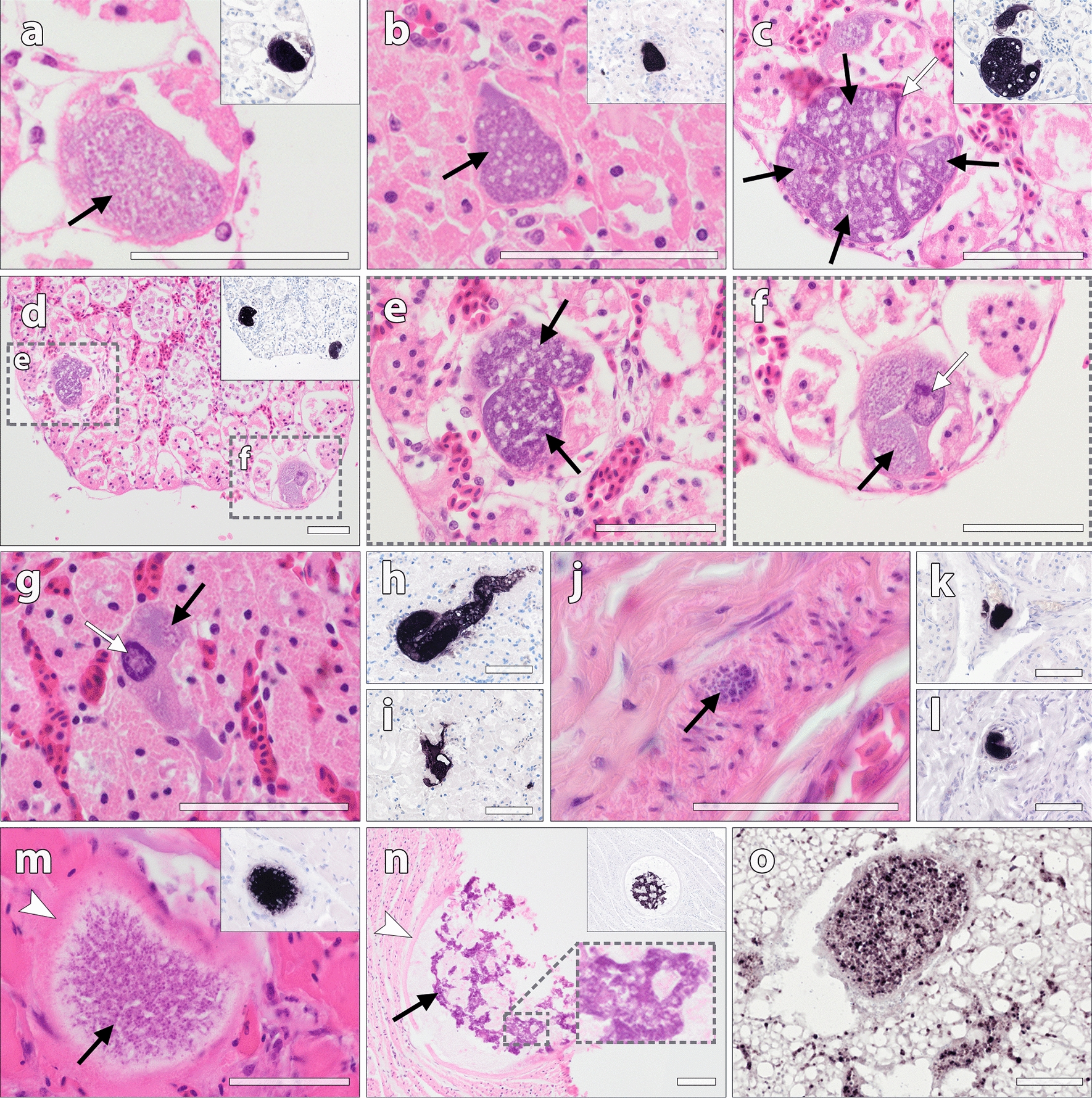
Parasite tissue stages of *Leucocytozoon*, *Haemoproteus* and *Plasmodium* in naturally infected wild birds, identified by CISH and in HE-stained sections.** a**–**f** Multiple meronts of *Leucocytozoon* sp. in the kidneys of a hawfinch coinfected with *Haemoproteus* and *Leucytozoon*. CISH using a *Leucocytozoon*-specific probe showed strong labelling of meronts in infected kidney cells, confirming their generic identity (inserts **a–d**). The meronts (black arrows) were mostly located in renal tubular cells and contained developing merozoites of varying maturity. Occasionally, a deformed or enlarged host cell nucleus, characteristic for *Leucocytozoon*-infected host cells, was visible (**c**, **f**, white arrows). **g–i** *Leucocytozoon* tissue stages in the kidneys of a hawfinch infected with the lineage HAWF7. The parasite stages were located in single infected host cells (**g**, black arrow), sometimes featuring an enlarged host cell nucleus (white arrow), or in multiple adjacent host cells (presumably renal tubule cells) (**h**), or in the intertubular space (**i**). **j–l** *Leucocytozoon* meronts in the proventriculus of an Eurasian bittern infected with *Leucocytozoon* sp. BOTSTEL01. **m** A *Haemoproteus* megalomeront in the pectoral muscle of a common chaffinch infected with *Haemoproteus* sp. CCF23, labelled by the *Haemoproteus*-specific probe (insert). The megalomeront contained numerous developing merozoites (black arrow), enclosed by an indistinct eosinophilic wall (white arrowhead). **n** A megalomeront detected in the muscular layer of the gizzard in a bullfinch infected with *Haemoproteus* sp. EMSPO03. The megalomeront was bounded by a thick eosinophilic wall (white arrowhead) and contained irregularly shaped, strongly basophilic cytomeres with developing merozoites (black arrow). **o** *Plasmodium* blood stages detected by CISH in the lung of a great tit co-infected with *P. relictum* SGS1 and *P. elongatum* GRW06. *Scale bars*: 50 μm

Concordant with the PCR results, CISH showed parasite stages in the histological samples of all 25 infected birds (Table 3). In most cases, detected CISH signals were confined to the lumen of blood vessels. As the intraluminal signals were relatively small (not larger than 5–6 μm) and endothelial meronts could not be observed in corresponding HE-stained sections, these stages were classified as blood stages of the parasites. In contrast to blood stages, parasite tissue stages were only observed in five birds (Table 3; Fig. 2).

**Table 3:** Haemosporidian parasites detected by CISH in bird carcasses collected in Austria from June to October 2020

Case ID	Host species	Parasite species & *cytB* lineages	Abundance of blood stages^a^	Distribution of tissue stages^a^
AH2017	*Carduelis carduelis*	*P. vaughani* SYAT05	+	–
AH2138	*Chloris chloris*	*Haemoproteus* sp. CARCHL01	+	–
AH2019	*Fringilla coelebs*	*Haemoproteus* sp. CCF23	++	–
AH2035	*Fringilla coelebs*	*Haemoproteus* sp. CCF23	+	–
AH2036	*Fringilla coelebs*	*Haemoproteus* sp. CCF23	+	–
AH2037	*Fringilla coelebs*	*Haemoproteus* sp. CCF23	++	Liver (meront) & pectoral muscle (megalomeront)
AH2038	*Fringilla coelebs*	*Leucocytozoon* sp. BRAM3	+	–
AH2049	*Fringilla coelebs*	*Haemoproteus* sp. CCF23, *Leucocytozoon* sp. BRAM3	+ (*H*, *L*)	–
AH2050	*Fringilla coelebs*	*Haemoproteus* sp. CCF23	+	–
AH2137	*Fringilla coelebs*	*Haemoproteus* sp. CCF23	+	–
AH2141	*Fringilla coelebs*	*P. relictum* GRW11	+	–
AH2024	*Coccothraustes coccothraustes*	*Haemoproteus* sp. HAWF6, *Leucocytozoon* sp. HAWF7, *Leucocytozoon* sp.COCCOC01	+ (*H*, *L*)	Kidney (meronts) (*L*)
AH2025	*Coccothraustes coccothraustes*	Leucocytozoon sp. HAWF7, *Leucocytozoon* sp. COCCOC01, *Leucocytozoon* sp. COCCOC02	++	–
AH2034	*Coccothraustes coccothraustes*	*Leucocytozoon* sp. HAWF7	++	Kidney (meronts)
AH2023	*Pyrrhula pyrrhula*	*Haemoproteus* cf. *majoris* EMSPO03, *Leucocytozoon* sp. PARUS25, *Leucocytozoon* sp. BT2	++ (*H*), + (*L*)	Gizzard (megalomeront) (*H*)
AH2018	*Cyanistes caeruleus*	*Leucocyotzoon* sp. PARUS4, *Leucocytozoon* sp. CYACAE07	+	–
AH2033	*Cyanistes caeruleus*	*Leucocytozoon* sp. PARUS4	+	–
AH2043	*Cyanistes caeruleus*	*P. elongatum* GRW06	+	–
AH2011	*Parus major*	*P. elongatum* GRW06, *Leucocytozoon* sp. PARUS4	+ (*P*, *L*)	–
AH2022	*Parus major*	*P. elongatum* GRW06	+	–
AH2032	*Parus major*	*P. relictum* SGS1, *P. elongatum* GRW06	+	–
AH2039	*Parus major*	*P. relictum* SGS1, *P. elongatum* GRW06	+++	–
AH2140	*Turdus merula*	*P. vaughani* SYAT05	+	–
AH2149	*Turdus merula*	*P. vaughani* SYAT05	+	–
AH2147	*Botaurus stellaris*	*Leucocytozoon* sp. BOTSTEL01	+	Proventriculus, gizzard & intestine (meronts)

+: low; ++: moderate; +++: high number of blood stages detected

–: no tissue stages detected

^a^ Letters in brackets refer to CISH signals obtained with genus-specific probes (*H*: *Haemo18S*, *L*: *Leuco18S, P: Plasmo18S*)

Two of three hawfinches exhibited *Leucocytozoon* tissue stages in their kidneys (Fig. 2a–i). Overall, the abundance of tissue stages was low in both birds. The meronts were mostly located in renal tubules and varied in size and maturity of the developmental stages. In some of the infected host cells an enlarged cell nucleus, characteristic for *Leucocytozoon*, was visible (Fig. 2f, g). In other organs, meronts were not observed. *Leucocytozoon* parasite stages were also found in the Eurasian bittern infected with *Leucocytozoon* sp. BOTSTEL01. However, compared to plenty blood stages observed in diverse organs, only few meronts were found in the muscle layer of the gizzard and in the in the mucosa of proventriculus and intestine (Fig. 2j–l).

Two finches exhibited tissue stages of *Haemoproteus* spp. in their organs. In one of them, a common chaffinch infected with *Haemoproteus* sp. CCF23, few small meronts were found in the liver as well as a single megalomeront in the pectoral muscle (Fig. 2m). In the other bird, a bullfinch infected with *Haemoproteus* cf. *majoris* EMSPO03, a single megalomeront of approximately 200 μm was detected in the muscle wall of the gizzard (Fig. 2n). Examination of additional tissue sections did not reveal any further megalomeronts in these birds.

Among the *Plasmodium*-infected birds, tissue stages were scarce or absent. One of the great tits infected with *Plasmodium elongatum* GRW06, showed severe macroscopic enlargement of spleen, indicating pathological involvement. CISH revealed plenty *Plasmodium* stages in the spleen, however, due to autolysis, tissue stages could not be identified in corresponding HE-stained sections. In another great tit, co-infected with *P. elongatum* GRW06 and *P. relictum* SGS1, numerous blood stages were observed in the vasculature, indicating heavy parasitaemia (Fig. 2o). To resolve the identity of the detected parasite stages in this case, CISH was repeated using species-specific probes for *P. elongatum* and *P. relictum*, revealing positive signals with both probes (Additional file 2). In none of the *P. elongatum*-infected birds, parasite stages were found in haematopoietic stem cells in the bone marrow.

Apart from splenic enlargement in the *P. elongatum* GRW06-infected tit, no pathologic changes were observed in association with the detected tissue stages.

## Discussion

This study reports on haemosporidian infections in wild passerine birds from Austria collected via dead bird monitoring using a citizen science approach. This method of sampling was used for the first time in avian haemosporidian research. Based on previously reported virulent *Plasmodium* infections in Common blackbirds [50], the present study aimed to explore whether and to what extent other native bird species are affected by severe parasite burdens of pathogenic haemosporidian lineages, contributing to avian mortality.

The study showed that citizen science can be beneficial for obtaining carcasses of naturally infected birds within a reasonable time period. In contrast to exploring haemosporidian infections in living birds, the investigation of bird carcasses offers the possibility to address questions related to the exo-erythrocytic development of the parasites, which remains underinvestigated in wild birds. Particularly for *Haemoproteus* and *Leucocytozoon* species, current knowledge about their exo-erythrocytic life cycle remains fragmentary and requires additional research [51]. The examination of organ samples of infected birds not only allows the detection and morphological description of developing tissue stages but also histologic assessment of affected organs. This is particularly important for determining infection severity and pathogenicity of haemosporidian species or lineages in different host species and better understanding the pathogenesis of Haemosporida-associated diseases. While citizen science provides an ideal framework to collect samples for such investigations, the sampling outcome in this study was constrained by several factors related to organization of the survey. First, participation of citizen scientists greatly depended on the publicity of the survey. For example, the response quote of citizen scientists was great after the first announcement at the beginning of the survey, but decreased over the following weeks, demanding further advertisement to raise public awareness and motivate citizens to report bird mortalities. Second, not all reported bird carcasses were suitable for collection due to their advanced decomposition or the lack of possibilities by the citizen scientists to store the collected birds adequately. Although haemosporidian parasites can be detected by CISH even in less-well preserved tissues samples, advanced autolysis prevents their morphological assessment in histological sections. Third, for logistic reasons, dead bird collection was geographically restricted, limiting overall bird sample size. Thus, to maximize sampling outcome, good publicity and communication with the citizen scientists before and during the survey, as well as logistical aspects concerning adequate storage, handling and transportation of collected carcasses need to be considered in future studies applying citizen science for the purpose of bird carcass collection.

Another important aspect to consider when collecting dead birds for investigating haemosporidian infections is sampling bias towards certain bird hosts. This is particularly critical with respect to comparing infection rates or apparent prevalences of haemosporidian parasites in bird carcasses to studies investigating living birds, and drawing conclusions about their pathogenicity. In the present study, dead bird sampling was strongly biased towards fringillid species. While species abundance certainly played a role here, this sampling bias can be explained by high mortality rates due to finch trichomonosis, an emerging infectious disease with high seasonality in mid-late summer. This protozoal disease primarily affects green finches, but also other finch species, albeit much less frequently [58], and has spread through Europe within the last decade, including Austria [59]. For this reason, the collection of green finch carcasses was restricted to fatalities not indicative for trichomonosis based on the citizen scientists’ reports (e.g. reported lethargy, ruffled plumage in combination with dysphagia). Yet, the pathological examinations revealed, that most of the collected finches had macroscopic lesions consistent with *T. gallinae* infection, indicating that these birds probably suffered from trichomonosis.

Keeping in mind its limitations and potential biases regarding bird collection, citizen science could present a complementary tool to study haemosporidian parasites in birds. Apart from generating scientific data, the involvement of citizen scientists in bird carcass collection also increases public awareness about avian diseases and bird health, which can be beneficial for engagement in bird conservation projects and general greater interest in science.

Including 81 birds of 24 passerine and non-passerine species, this investigation showed haemosporidian parasites in 31% of the birds, however, the proportion of infected birds varied considerably across host species. With finches and tits dominating in numbers, the majority of the infected birds presented low parasite burdens in both blood and tissues. Particularly tissue stages of *Haemoproteus* and *Leucocytozoon* parasites have been reported to cause haemorrhages and necroses or provoke inflammatory processes [7, 13, 50, 60], however, in none of the cases investigated here, tissue reactions were associated with the detected meronts or megalomeronts, suggesting an overall minor role of these parasites for the bird fatalities. Instead, most bird deaths probably resulted from other causes, most of all traumatic injuries and fatal infections with *Trichomonas* parasites (Additional file 1). However, two great tits clearly showed signs of avian malaria, indicating detrimental effects of the involved *Plasmodium* parasites. One of the two birds exhibited marked parasitaemia as demonstrated by abundant CISH signals in the blood, whereas the other had numerous parasite stages in the spleen, associated with severe splenic enlargement. Although it remains questionable whether avian malaria was more than a contributory factor for death of the birds, these two cases are particularly interesting, as both featured infections with *P. elongatum* GRW06, a generalist parasite rarely documented in species of the Paridae so far. This parasite infects numerous avian species of different families worldwide (MalAvi database, [2]) and can be highly virulent for non-adapted hosts [61–63]. However, with only two reports of GRW06 in a great tit [57] and blue tit [64] from Austria, records from the family Paridae are virtually absent. Notably, in the present study, GRW06 was found in four of ten great tits and one of three blue tits. The positive CISH results with the *P. elongatum*-specific probe detecting parasite stages in the tissues suggest that this parasite is able to develop in Paridae species. However, assuming that tits are susceptible to *P. elongatum* GRW06, it remains difficult to explain why *P. elongatum* GRW06 has been absent in free-living conspecifics sampled in other European localities, despite its prevalence in different sympatric passerines [65–67]. An experimental study showed that certain songbirds, specifically common starlings, are resistant to GRW06 infection [68]. Similar to starlings, innate resistance of tits would explain the absence of GRW06 in these birds. However, given the detected parasite stages in the tits from this study, innate resistance could hardly explain the lack of GRW06 records in Paridae. It has to be noted, that there is a slight chance, that the CISH signals detected in the *P. elongatum* positive tits from this study could also represent sporozoites, the presence of which does not necessarily imply successful infection and thus susceptibility of the birds. In order to confirm the findings and to prove susceptibility of tits to *P. elongatum* GRW06, experimental studies are needed.

Aside from tits, the results showed high infection rates in finches, consistent with findings from other studies investigating living birds [69, 70]. Accordingly, most lineages detected in the finches here were previously recorded in this host family, except for *Plasmodium vaughani* SYAT05 and *Leucocytozoon sp.* BT2, found in a goldfinch and a bullfinch, respectively. *Plasmodium vaughani* SYAT05 typically infects species of Turdidae, most of all blackbirds [1, 3, 50], however, it has also been recorded in non-Turdidae hosts, including common chaffinches [71, 72]. While infections of finches with SYAT05 seem to be the exception, the parasite stages detected in the blood of the goldfinch suggest complete parasite development in this host species. The same cannot be concluded definitely for the *Leucocyotzoon* lineage BT2 detected in the bullfinch, as this bird was co-infected with another *Leuocytozoon* lineage, hampering attribution of the observed parasite stages to either one of the two lineages. However, although BT2 was primarily found in species of Muscicapidae, Phylloscopidae and Sylvidae, it was also recorded in other Fringillidae species [73, 74], providing support for the occurrence in this bird family.

In addition to blood stages, CISH revealed parasite tissue stages in some birds, contributing new data on the exo-erythrocytic development of *Haemoproteus* and *Leucocytozoon* lineages in passerines. In hawfinches, *Leucocytozoon* meronts were exclusively found in the kidneys, suggesting this organ to be a common site of exo-erythrocytic development of the detected lineages, consistent with descriptions of renal merogony in other passeriform-specific *Leucocytozoon* [51, 75–77]. In the hawfinch co-infected with two *Leucocytozoon* lineages (HAWF7 and COCCO01), multiple meronts were distributed across the renal parenchyma and seemed to develop primarily in epithelial cells of tubules, sometimes leading to expansion of the infected cells, occupying most of the tubular lumen. While meronts found in neighbouring tubular cells equalled in size and maturity, meronts located in distant renal tubules showed varying stages of maturity, suggesting overall asynchronous renal merogony of the parasites. However, as the observed tissue stages in this bird could not be assigned to one of the two detected *Leucocytozoon* lineages, lineage-dependent developmental differences cannot be excluded.

As the results showed, findings of exo-erythrocytic merogony of *Haemoproteus* parasites were restricted to single megalomeronts detected in the gizzards of a chaffinch infected with *Haemoproteus* sp. CCF23 and a bullfinch infected with *Haemoproteus* sp. EMSPO03. Both lineages were previously recorded in finches [58, 69, 70, 78], but have not been morphologically described yet. Megalomeronts have only been documented in nine *Haemoproteus* species so far, and knowledge about the patterns of *Haemoproteus* exo-erythrocytic development within different host species remains fragmentary [51, 79]. In several reports, the formation of megalomeronts were associated with abortive infections in aberrant hosts [8, 10, 13, 51, 60, 80]. However, recent studies demonstrated *Haemoproteus* megalomeronts in naturally infected passerines, proposing that they are regular stages during exo-erythrocytic development [79, 81]. The findings of the present study support this suggestion, as megalomeronts were found along with erythrocytic stages of the parasites. Notably, the megalomeront found in the bird infected with *Haemoproteus* sp. EMSPO03 strongly resembled megalomeronts of three closely related lineages of *H. majoris* (PHSIB1, PARUS1, PHYBOR04), sharing the same unique morphological characters such as irregularly formed cytomeres and a thick wall [79, 81]. Based on the morphological similarities, this study suggest that EMSPO03 also belongs to the *H. majoris* species complex, supporting previous suggestions, that closely related lineages have similarly developing megalomeronts [81]. While EMSPO03 still requires morphological characterization, phylogenetic analyses corroborate its close relationship to other *H. majoris* lineages [79, 82]. Interestingly, in all three other lineages (PHSIB1, PARUS1, PHYBOR04), megalomeronts were particularly numerous in the kidneys of the infected birds, whereas in the finch infected with EMSPO03, only a single megalomeront was found in the gizzard, indicating differences concerning the site of development, which might be a function of the host-lineage combination. Further investigations are needed to elucidate patterns of exo-erythrocytic merogony of *H. majoris* lineages.

## Conclusions

While the findings of this study suggest generally little contribution of haemosporidian infections to morbidity of investigated bird hosts, there are indications that certain *Plasmodium* lineages at least play a role in overall clinical manifestation, either as main causes or as concurrent disease agents. In order to be able to assess the significance of these infections for particular host species, such as the tits in this study, additional investigations including larger bird sample sizes are inevitable. The present study demonstrates that citizen science can be applied to prospectively collect carcasses of target passerine species, which do find their way into pathological archives less frequently. This approach opened the opportunity to study pathological effects of haemosporidian infections and gain knowledge about exo-erythrocytic development of haemosporidian lineages naturally occurring in passerines.

## Supplementary Information

**Additional file 1.** Case details for all bird carcasses confirmed positive for haemosporidian parasites by PCR and sequencing.

**Additional file 2.** Parasite stages of *Plasmodium relictum* SGS1 and *P. elongatum* **GRW06 **detected in a brain section of a coinfected great tit (*Parus major)* using CISH and species-specific probes. a Blood stages of *P. relictum* SGS1 in the lumen of a brain vessel, labeled by the *P. relictum*-specific probe (Prel18S). b Parasite stages of *P. elongatum* GRW06 in another brain vessel of the same infected individual, labeled by the *P. elongatum*-specific probe (Pelo18S).

## Data Availability

The dataset supporting the conclusions of this article is included within the article and its additional files.
