# Supplementary figures and images for: A citizen science-based survey of avian mortality focusing on haemosporidian infections in wild passerine birds
Source: Malar J. 2021 Oct 23;20:417. doi: 10.1186/s12936-021-03949-y (PMC8542282; doi:10.1186/s12936-021-03949-y)

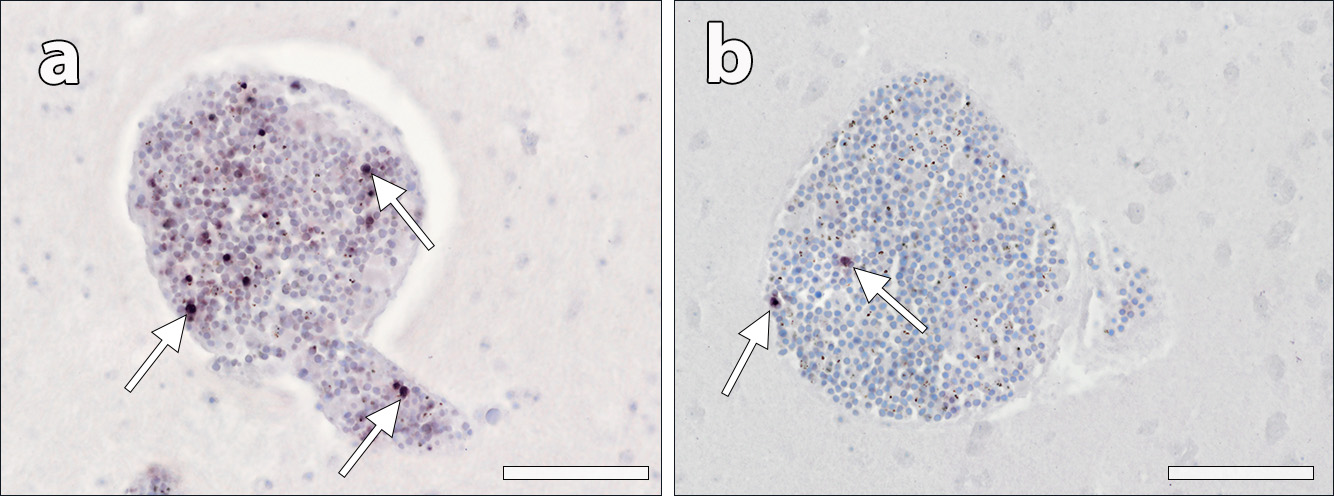

Supplement: Supplementary file 2 — Additional file 2. Parasite stages of Plasmodium relictum SGS1 and P. elongatum GRW06 detected in a brain section of a coinfected great tit (Parus major) using CISH and species-specific probes. a Blood stages of P. relictum SGS1 in the lumen of a brain vessel, labeled by the P. relictum-specific probe (Prel18S). b Parasite stages of P. elongatum GRW06 in another brain vessel of the same infected individual, labeled by the P. elongatum-specific probe (Pelo18S). [file 12936_2021_3949_MOESM2_ESM.jpg]
